# Magnetron Sputtering Formation of Germanium Nanoparticles for Electrochemical Lithium Intercalation

**DOI:** 10.1002/cphc.202400594

**Published:** 2024-11-08

**Authors:** Tommaso Pajola, Anika Padin, Benjamin E. Blowers, Francesca Borghi, Alessandro Minguzzi, Emiliano Bonera, Alberto Vertova, Marcel Di Vece

**Affiliations:** ^1^ Interdisciplinary Centre for Nanostructured Materials and Interfaces (CIMaINa) and Physics Department “Aldo Pontremoli” Università degli Studi di Milano Via Celoria 16 20133 Milan Italy; ^2^ Dipartimento di Chimica Università degli Studi di Milano Via Golgi 19 20133 Milan Italy; ^3^ Dipartimento di Scienza dei Materiali Università di Milano-Bicocca Via Cozzi 55 20125 Milan Italy

**Keywords:** Germanium, Lithium, Nanoparticles, battery, Raman spectroscopy

## Abstract

In the drive towards increased lithium based battery capacity, germanium is an attractive material due to its very high lithium storage capacity, second only to silicon. The persistent down‐side is the considerable embrittlement accompanying its remarkable volume expansion of close to 300 %. A proven method to accommodate for this lattice expansion is the reduction of the size towards the nanoscale at which the fracturing is prevented by “breathing”. In this work we employed a novel magnetron sputtering gas aggregation nanoparticle generator to create unprecedented layers of well‐defined germanium nanoparticles with sizes below 20 nm. The electrochemical lithium intercalation was monitored by a suite of techniques under which Raman spectroscopy, which provided clear evidence of the presence of lithium inside the germanium nanoparticles. Moreover, the degree of lattice order was measured and correlated to the initial phases of the lithium‐germanium alloy. This was corroborated by electron diffraction and optical absorption spectroscopy, of which the latter provided a strong dielectric change upon lithium intercalation. This study of low lithium concentrations inside layers of well‐defined and very small germanium nanoparticles, forms a new avenue towards significantly increasing the lithium battery capacity.

## Graphical abstract

Lithium was electrochemically inserted into Germanium nanoparticle assembled thin films functioning as anode, potentially exceeding the current lithium battery capacity significantly. The Germanium nanoparticles were fabricated with a magnetron sputtering gas aggregation nanoparticle generator. The lithium concentrations were followed by Raman spectroscopy while optical absorption spectroscopy demonstrated dielectric changes. This work provides a feasible method to improve lithium battery performance.

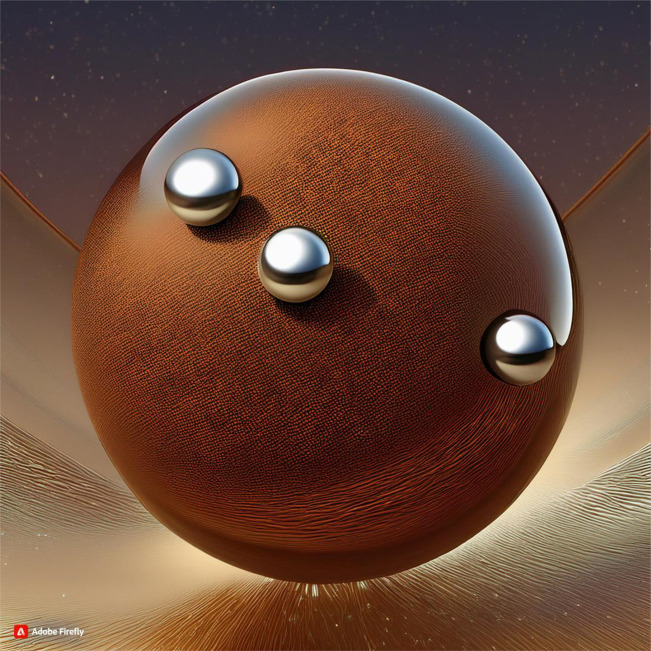

## Introduction

Lithium based batteries have become ubiquitous due to their high energy storage capacity, fast (de)charging and considerable cyclability lifetime.^[1]^ Improving these qualities even further will be highly beneficial and therefore warrants the exploration of alternative approaches.^[2]^ The most common lithium batteries use a graphite anode, which binds lithium to six carbon atoms, creating a LiC_6_ compound.^[3]^ The theoretical capacity of a graphite anode is limited to 372 mAh/g[^4^, ^5^, ^6^]; such limit has already been reached in current batteries, making further improvements in graphite anodes unfeasible. Silicon and Germanium have a much higher theoretical gravimetric capacity than graphite, 4200 mAh/g[^4^, ^6^] and 1620 mA respectively, making them very promising materials for battery applications. However, during charging, both materials undergo considerable volumetric expansion, respectively of 400 %^[7]^ and 370 %,^[8]^ enhancing embrittlement and electrode fading.^[6]^ To accommodate for the lattice expansion, particles which can “breathe” could have much longer lifetimes as they do not fracture, thus improving cycling stability of the battery.^[9]^ Although the slightly smaller lattice expansion of germanium already may be favourable for battery application, lithium has a diffusivity 400 times higher in germanium than in silicon,^[10]^ making germanium more attractive for high power rate electrodes. Moreover, intrinsic germanium has a much higher electronic conductivity since the bandgap of germanium (0.66 eV at 300 K) is smaller than that of silicon (1.12 eV at 300 K).^[11]^ Despite receiving less attention, germanium has a very high volumetric capacity (7366 AhL^−1^ for Li_15_Ge_4_), unmatched except for silicon (8334 AhL^−1^ for Li_15_Si_4_).[^12^, ^13^, ^14^, ^15^, ^16^]

Various approaches to create nano‐ or microscale silicon nanostructures, such as for example columnar amorphous‐silicon film^[17]^ and nano‐vaults,^[18]^ have been used to improve the lithium interaction for batteries. Large germanium nanoparticles and their porous structures for lithium battery purposes have been fabricated with a wide range of techniques such as laser pyrolysis[^5^, ^19^] and chemical synthesis.[^20^, ^21^, ^22^] Reducing the scale of the germanium nanoparticles even further, towards the order of magnitude of 10 nm or smaller is required to optimize the accommodation of lithium induced stress and therefore preventing electrode embrittling. Micrometer sized particles are not small enough to prevent embrittlement and therefore here we studied nanometer sized germanium particles for lithium storage. In this work we use the novel method of a magnetron sputtering gas phase aggregation nanoparticle generator,[^23^, ^24^, ^25^] with which layers of well‐defined nanoparticles with sizes around 10 nm can be routinely fabricated.[^26^, ^27^] A suite of techniques was used to characterize the germanium nanoparticles post electrochemical lithium intercalation at different (low) lithium concentrations.

## Experimental

**Figure 1:**
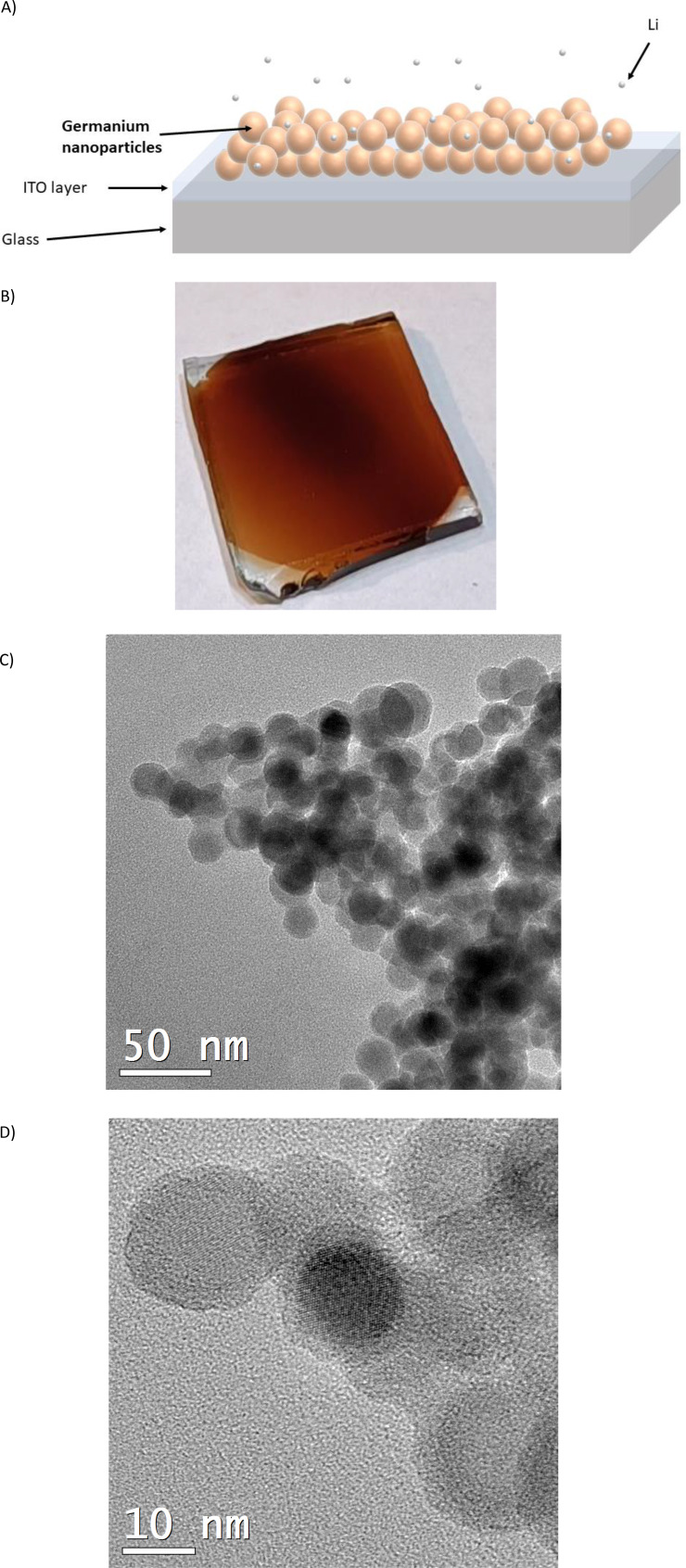
A) Schematic cartoon of Germanium nanoparticles on top of ITO during lithium exposure. B) Photograph of typical germanium nanoparticle assembled thin film of ITO on glass (~1 cm^2^). TEM micrographs of Germanium nanoparticles from a C) pristine and D) 20 % lithiated sample (main lattice distance 3.19 Å).

Gas aggregation magnetron sputtering nanoparticle generators are routinely used to produce nanoparticles with good control over the properties, such as size and composition.[^23^, ^24^, ^25^] The germanium nanoparticles in this work were deposited with such a device (NC200U−B Oxford Applied Research Ltd.)^[23]^ at room temperature from an Antimony n‐doped germanium target on an ITO on glass substrate (1 cm^2^) as schematically depicted in Figure 1a and with a representative example photograph in Figure 1b. The magnetron power was ~130 W (0.4 A and 330 V) with argon gas (99.999 % purity) used both as plasma sputter and carrier gas inside the gas aggregation chamber with a flow rate of 20 sccm. The operation pressure inside the reaction and deposition chambers were ~10^−3^ and ~10^−4^ mbar, respectively. After fabrication the samples were stored in air because the native oxide layer protects against further oxidation.

The electrochemical intercalation of lithium into germanium nanoparticles was achieved with the three‐electrode cell and a solution of propylene carbonate with lithium nitrate salt (LiNO_3_) dissolved to saturation as electrolyte. Due to lithium's vigorous reactions with oxygen and water vapor, the measurements were conducted in a sealed glovebox with a pure argon atmosphere. A Palm Sense potentiostat was used, specifically designed for electrochemistry, capable of performing Open Circuit Potential (OCP) and Cyclic Potentiometry (CP). The current densities used varied between 0.1 till 0.74 μA/cm^2^ with an exposed surface area of 0.64 cm^2^ and loading times between 1 and 4 hours. Because the minute germanium QD layer thickness modulations only have a very small effect on the electric resistance of the layer, we did not observe any potential variation and therefore it is safe to conclude that there is no influence on the electrochemical lithium intercalation.

The TEM images were recorded with a FEI Tecnai F20, with an accelerating voltage of 200 kV on a S‐Twin lens providing a point resolution of 0.24 nm. An energy dispersive X‐ray spectrometer (EDS) with ultrathin window (Xplore‐Oxford Instruments) was used for elemental analysis, which confirmed the presence of germanium in the nanoparticles. The germanium nanoparticles were scraped from the sample with a metal knife, dispersed in isopropanol by sonication and then drop casted on an a‐C TEM grid.

The surface morphology and the root‐mean‐square roughness of the germanium nanoparticle assembled film was determined by atomic force microscopy (AFM) using a Multimode AFM by Bruker operated in Tapping Mode in air, equipped with standard probes with radius below 10 nm. AFM raw images were flattened by subtracting a paraboloid to remove the scanner‐induced bow and the sample tilt. A median filter was applied to AFM images to remove high‐frequency noise.

UV‐visible absorbance spectra were recorded with a commercial double beam Agilent Cary‐100 spectrophotometer with a wavelength range from 200–800 nm and a wavelength resolution of 1 nm. From the optical absorbance the thickness of the germanium nanoparticle layers were estimated via the Beer‐Lambert law,[^28^, ^29^] which ranged from 29–119 nm, due to flux variations during deposition. From the estimated germanium layer thickness, the lithium concentration was calculated in order to set the applied current and the intercalation time.

Raman measurements were performed with a Jobin‐Yvon T64000 spectrometer with an excitation wavelength of 532 nm and an excitation power of about 1 mW to avoid heating of the sample. The spectra were collected with a 100×0.90NA objective.

## Results and Discussion

**Figure 2:**
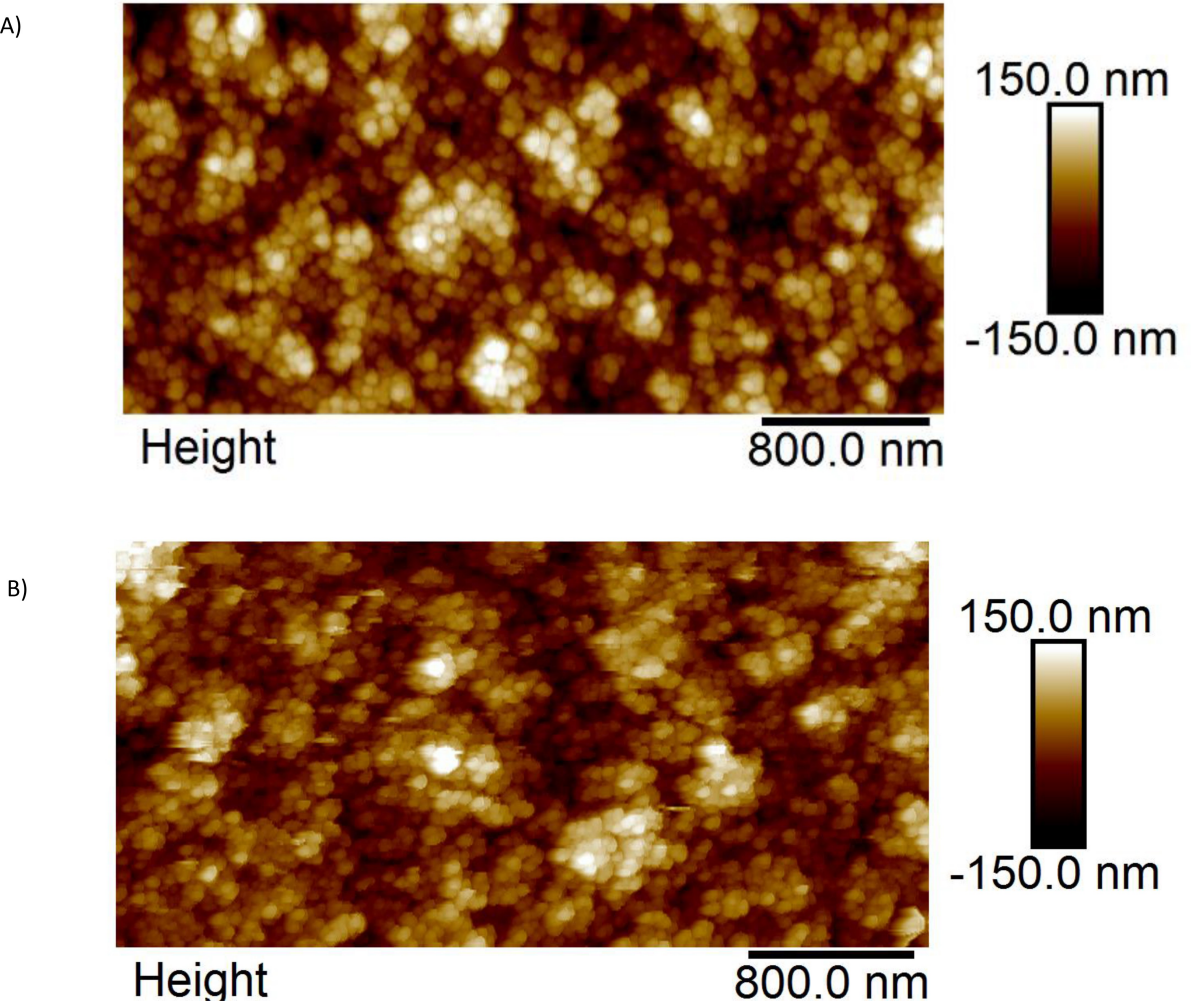
AFM measurements on Germanium nanoparticle assembled films for A) pristine and B) 20 % Li‐loaded sample. The lateral information is convoluted with the AFM tip, resulting in larger visible size. The AFM image of the 20 % Li loaded sample in B) is more noisy due to the presence of electrolyte residues.

The germanium nanoparticles were deposited with identical conditions which yielded slightly different thicknesses due to the nanoparticle generator's inherent flux fluctuations. After the deposition of germanium nanoparticle assembled layers on the ITO and subsequent lithiation, the nanoparticles were extracted and characterised by TEM. The TEM imaging of the pristine germanium nanoparticles shows a clear size of about 15 nm and often high crystallinity as shown in Figure 1c‐d. Furthermore, it is clear that the germanium nanoparticles adhere strongly to each other as often clusters of particles were observed. The AFM characterisation of the pristine and 20 % lithium concentration germanium nanoparticle assembled thin film, shown in Figure 2a‐b respectively, confirms both the size of about ~15 nm (as obtained by the height profiles). The germanium nanoparticles were confirmed to be stable because they retained a spherical shape after 20 % lithiation, no fractured germanium is visible, and no fragments were found.

**Figure 3:**
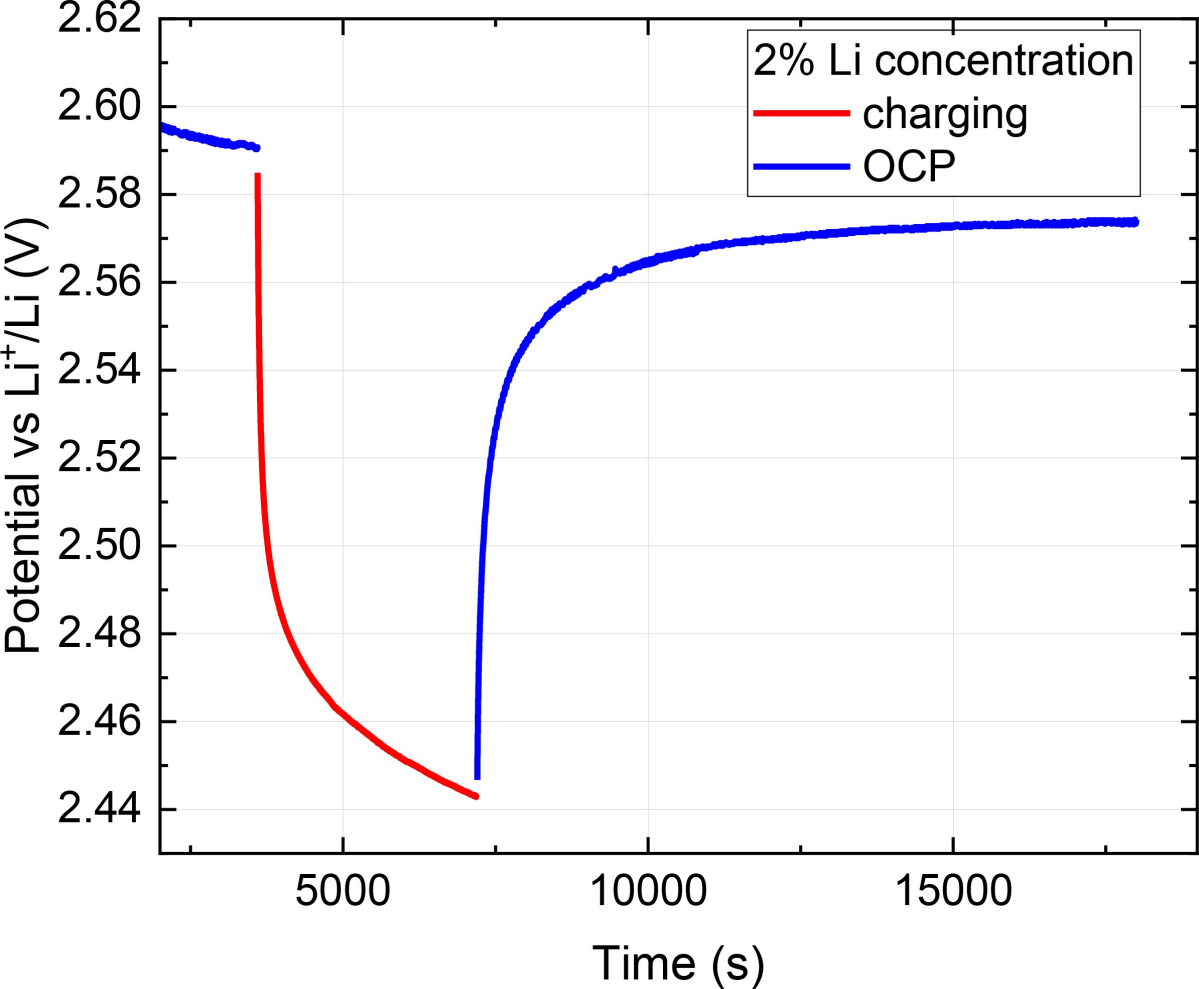
Electrochemical lithium charging pulse to the germanium nanoparticles electrode, showing the overpotential accompanying the applied current of 0.18 μA/cm^2^ aiming at 2 % lithium concentration.

The electrochemical intercalation of lithium was achieved by applying a potential to the germanium nanoparticles electrode, which was exposed to the lithium containing solvent. At the beginning of the charging process, the potential rapidly decreases from 2.60–2.44 V due to the required overpotential accompanying the applied current (Figure 3). After a charging pulse, the open circuit potential increases in about 7500 s to a lower plateau voltage of 2.57 V, as compared to before the lithiation pulse (2.60 V), due to successful lithium intercalation into the crystalline structure of the germanium nanoparticles. The potential corresponds to the lithium concentration via the Nernst equation,^[30]^ with an adjustment for the double layer^[31]^ capacitance.

Although a surface oxide shell on the air‐exposed germanium nanoparticles is expected,[^32^, ^33^] during the first cathodic polarization, the germanium oxide is reduced with the formation of germanium metal.^[34]^ The potential recovery time of 7500 s after the current is halted, likely due to the diffusion of lithium from the outer layers to the inner parts, by which the lithium concentration at the surface is decreasing, leading to a higher electrical potential. The diffusion coefficient *D* for lithium inside germanium at room temperature^[35]^ is 5×10^−5^ cm^2^ s^−1^, which provides a diffusion time to cross a 60 nm germanium layer of approximately 1 μs, by using Fick's approximation *t*=*L* ^2^/*D*, with *t* the diffusion time, and *L* the film thickness.^[36]^ In contrast to a continuous thin film, the porous nature of the germanium nanoparticle assembled film studied in this work inhibits the diffusion considerably due to the limited surface area of the nanoparticle connections and explains the much longer time required in this experiment.

**Figure 4:**
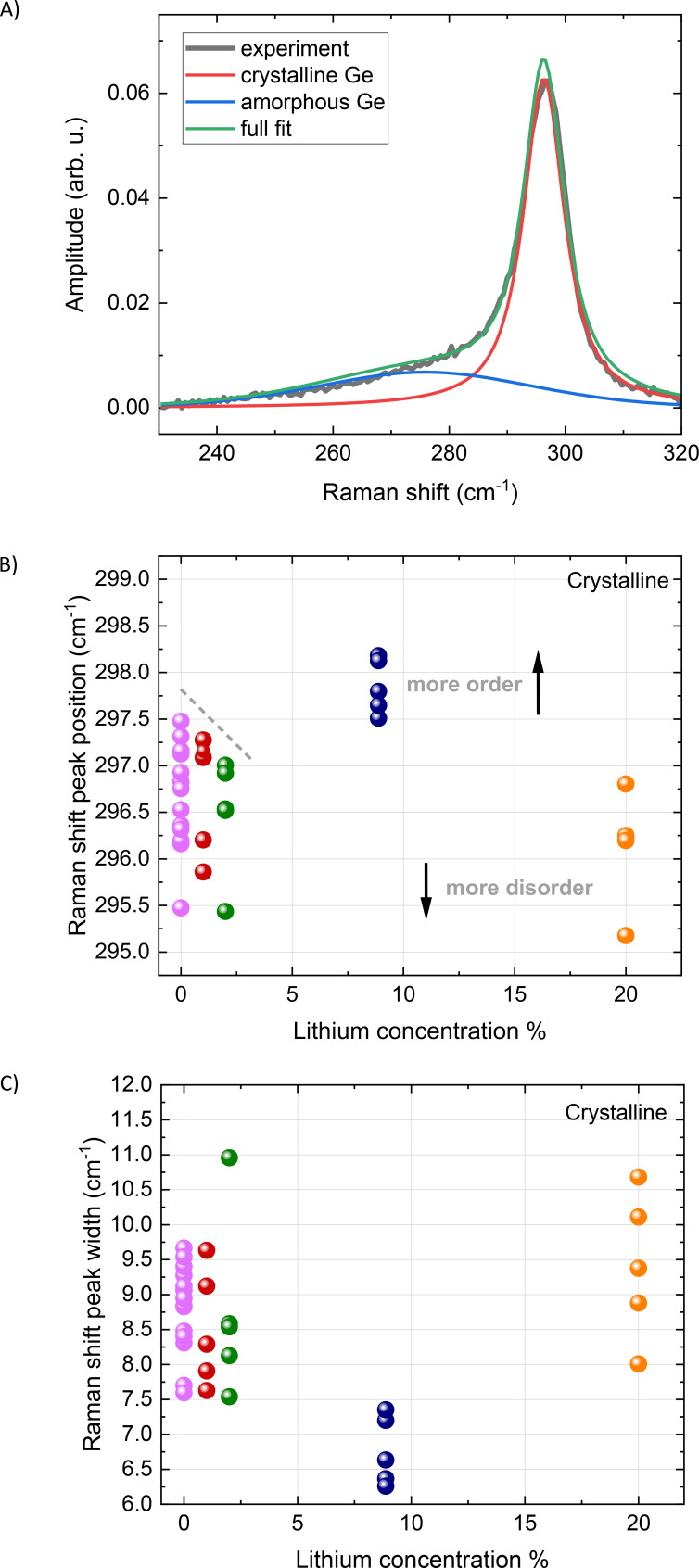
A) Raman shift peak including fitting contributions. Raman peak shift B) position and C) width of the crystalline fraction as function of lithium concentration for pristine and lithiated germanium nanoparticle assembled thin films. Each point at each concentration correspond to a different measuring position on the samples. The dashed lines are a guide for the eye, indicating crystallisation on the 8.8 % sample and reduction of crystallization in the 20 % sample.

The lithiated germanium nanoparticle layers were also investigated by Raman spectroscopy, which yielded the germanium Raman peak shift and width for the crystalline fraction as a function of lithium concentration as shown in Figure 4a–c. The spectra were fitted with a crystalline and an amorphous band, providing good quality of the fitting as can be seen in Figure 4a and in good agreement with literature.^[37]^ The combined presence of both crystalline and amorphous germanium has been observed before with the same nanoparticle deposition method.^[27]^ The ratio between the amplitude of the amorphous and crystalline bands of the germanium nanoparticle layers was around 20±6 % with the exception of the 8.8 % lithium concentration where it was about 11±3 % (SI, Figure 1). The amplitude ratio only provides insight into the relative concentration differences because it is not possible to extract a concentration ratio from the Raman measurement due to unknown efficiency values for each phase. The Raman peak shift position as seen in Figure 4b shows that the pristine sample varied between 295.5–297.5 cm^−1^ for measurements on different positions on the sample with a 0.5 μm focus size, which indicates slightly different germanium crystallinity at different positions on the sample. On going from 1–8.8 % lithium concentration, the Raman peak position spread reduces significantly and becomes more concentrated, likely due to lithium intercalation induced atomic rearrangement. The Raman shift peak positions for 1 % and 2.2 % lithium concentration are more localised around 296.5 cm^−2^, close to the pristine values but with less spread as indicated by the dashed lines in Figure 4b, indicating the onset of crystallisation. It should be noted that the amorphous/crystalline ratio depends sensitively on many of the deposition parameters such as for example pressure and aggregation distance, which has not been explored in this work.

A shift towards higher wavenumbers can also be an indication of an increase in crystallinity, such as the reduction of defects, disorder, likely due to an incomplete phase transition. The Raman shift peak significant movement to 298.0 cm^−1^ at 8.8 %, therefore it is compatible with less disorder or change in strain state in the germanium nanoparticles, while the shift below 297.0 cm^−1^ at 20 % lithium concentration, points to a transition towards a more disordered phase, or a higher tensile strain crystal, probably caused by the formation of defects due to the excessive intercalation of lithium. Electrochemical lithium intercalation in evaporated germanium thin films shows indeed a phase evolution from amorphous to cubic Li_15_Ge_4_.^[16]^ A range of solid crystalline compounds can be formed including LiGe, Li_11_Ge_6_, Li_15_Ge_4_, and Li_22_Ge_5_ ^[38]^ with each different crystal structures as also determined by x‐ray diffraction on germanium nanoparticles.^[5]^ The crystal phases correspond to different electrochemical potentials.^[39]^

The width of the Raman shift peaks for the crystalline fraction in Figure 4b can be attributed to a similar trend as the peak position. The pristine germanium nanoparticles exhibit a large spread in the width of the Raman shift peak, while at initial low concentrations of 2.2 % and 8.8 % the width becomes narrower after which it slightly increases again. The analysis of the reduced width also suggests that the 8.8 % lithium concentration appears with a higher crystal order. This order is reduced when we move to the maximum lithium intercalation of 20 %, which exhibits broader bands. A similar analysis for the fitting results of the amorphous part of the peak is not possible due to the high uncertainty.

**Figure 5:**
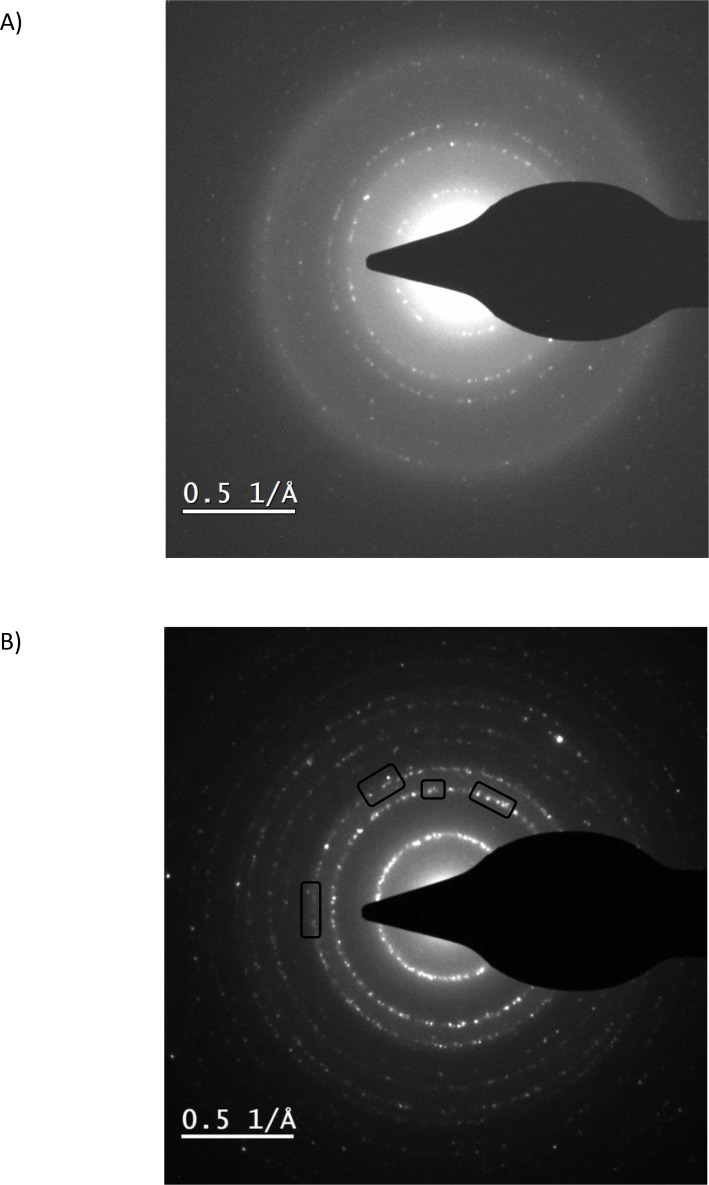
Electron diffraction patterns of A) pristine sample and B) 20 % Li‐loaded sample with a splitting of the pattern as indicated by black lined boxes. The crystal splitting indicates pockets of different crystal phases with different lattice distances.

The pristine germanium nanoparticle crystal planes were measured by electron diffraction as shown in Figure 5a, which provided 3.15, 1.93 and 1.64 Å, in good agreement with the germanium bulk crystal lattice parameters^[40]^ within the scope of lattice contractions due to the nanoscale.^[41]^ The 2 % and 20 % lithium (Figures 5b) loaded samples had significantly increased lattice parameters of 3.23, 1.98, 1.69 and 3.19, 1.97, 1.68 Å, respectively, likely due to the presence of lithium inside the germanium crystal lattice, causing an expansion in the order of 2 %. This is confirmed by the electron diffraction of Figure 1d, which provides a main crystal plane distance of 3.19 Å. It is clear that the lithium at all concentrations in this work did not embrittle the nanoparticles because in line with the AFM results, in the TEM images of the post‐lithiation germanium nanoparticles no fractured particles were detected and the nanoparticles have a shape and size comparable to the pristine nanoparticles.

**Figure 6:**
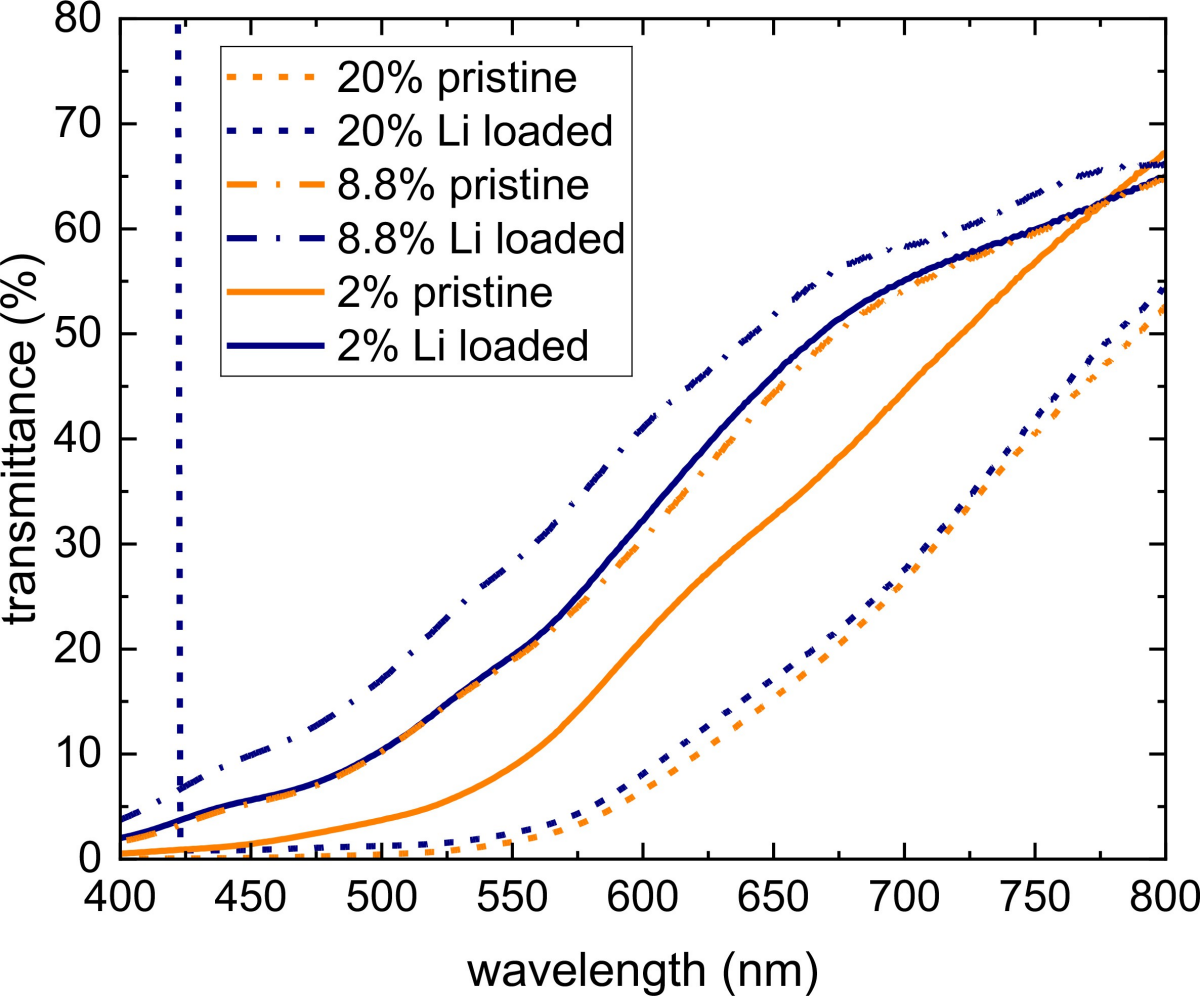
Optical transmission measurements of 2, 8.8 and 20 % lithium loaded germanium nanoparticle layers, exhibiting clear blue shifts after lithium absorption. The could be caused by electronic changes or increased quantum confinement by reduced germanium nanoparticle size.

The post‐lithiation optical transmission measurements revealed a marked change upon lithiation of the germanium nanoparticle assembled film for every concentration (Figure 6). A clear blue shift of about 50 nm and a few nm for the 2 % and 20 % lithium concentrations, respectively, not only confirms the presence of lithium inside the germanium nanoparticles, but also indicates a strong electronic interaction, affecting the optical properties of the germanium nanoparticles. A blue shift in transmission indicates an increased germanium band gap, which could be the result of lattice (local) compression, electronic interactions between host and intercalating species (Li), or increased quantum confinement by a reduction of the germanium nanocrystal size. Such striking optical changes in thin metal films with the intercalating species being hydrogen has been reported before and assigned to changes in the density of states.[^42^, ^43^] The smaller blue shift for the 20 % lithium concentration is likely due to the thicker germanium nanoparticle film thickness, reducing the overall optical transmission and therefore also the transmission changes.

Because the lithium diffusion in the germanium nanoparticle assembled film is inhibited by the presence of “bottlenecks” at the nanoparticle connections, it cannot be excluded that the surface layers have a higher lithium concentration with respect to the lower layers. This could therefore induce structural phase transitions at the upper lying germanium nanoparticles while the bottom nanoparticles are lagging behind. This could explain why the Raman measurements, which are particularly sensitive to the first few nm, show strong effects, while the electron diffraction provides a rather small lattice expansion.

Concluding, germanium nanoparticle assembled thin films have been successfully fabricated on conductive substrates for electrochemical lithium intercalation. The presence and effect of lithium in the germanium nanoparticles was confirmed by TEM, Raman spectroscopy and optical absorption spectroscopy. Raman spectroscopy provided detailed information about the presence of structural changes within the germanium‐lithium compound, which included the presence of phase transitions, corroborated by electron diffraction. The presence of lithium within the germanium crystal lattice affects the electronic properties of germanium, here resulting in a change in the optical absorption behaviour, which forms a new tool to investigate lithiation. This work forms a first, low lithium concentration, step towards higher capacity batteries based on germanium nanoparticles produced by magnetron sputtering.

## Supporting Information Summary

Amorphous‐crystalline ratio from Raman shift peak fitting.

## Author Contributions

MDV conceived and supervised the work. AV and AM supervised the electrochemistry experiments. TP, AP and BEM performed the experiments. FB performed the AFM measurements and EB performed the Raman experiments and analysis. MDV fabricated the samples and wrote the manuscript. All authors discussed and edited the manuscript.

## Conflict of Interests

The authors declare no conflict of interest.

1

## Supporting information

As a service to our authors and readers, this journal provides supporting information supplied by the authors. Such materials are peer reviewed and may be re‐organized for online delivery, but are not copy‐edited or typeset. Technical support issues arising from supporting information (other than missing files) should be addressed to the authors.

Supporting Information

## Data Availability

The data that support the findings of this study are available from the corresponding author upon reasonable request.
