# Supplementary material for: Magnetron Sputtering Formation of Germanium Nanoparticles for Electrochemical Lithium Intercalation
Source: Chemphyschem. 2024 Nov 8;26(1):e202400594. doi: 10.1002/cphc.202400594 (PMC11747581; doi:10.1002/cphc.202400594)
Supplement: Supplementary file 1 — Supporting Information [file CPHC-26-e202400594-s001.pdf]

ChemPhysChem

Supporting Information

# Magnetron Sputtering Formation of Germanium Nanoparticles for Electrochemical Lithium Intercalation

Tommaso Pajola, Anika Padin, Benjamin E. Blowers, Francesca Borghi, Alessandro Minguzzi, Emiliano Bonera, Alberto Vertova, and Marcel Di Vece*

# Supporting Information

# Magnetron sputtering formation of Germanium nanoparticles for electrochemical Lithium intercalation

Tommaso Pajola[1], Anika Padin[1], Benjamin E. Blowers[1], Francesca Borghi[1], Alessandro Minguzzi[2], Emiliano Bonera[3], Alberto Vertova[2] and Marcel Di Vece[1*]

[1] *Interdisciplinary Centre for Nanostructured Materials and Interfaces (CIMaINa) and Physics Department "Aldo Pontremoli", Università degli Studi di Milano, Via Celoria 16, 20133, Milan, Italy*

[2] *Dipartimento di Chimica, Università degli Studi di Milano, Via Golgi 19, 20133, Milan, Italy*

[3] *Dipartimento di Scienza dei Materiali, Università di Milano-Bicocca, Via Cozzi 55, 20125, Milan, Italy*

[*] *Corresponding author: marcel.divece@unimi.it*

Figure S1

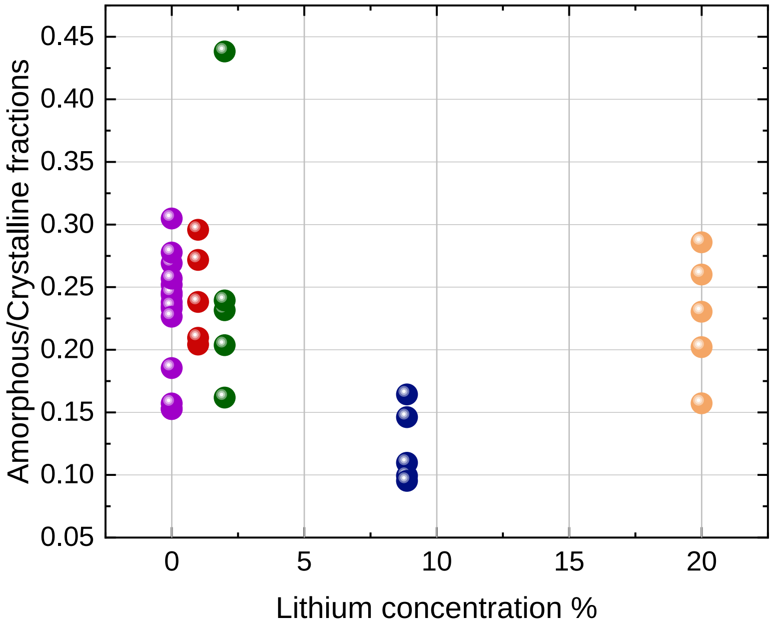


Amorphous-crystalline ratio from Raman shift peak fitting as function of lithium concentration. Each point at each concentration correspond to a different measuring position on the samples.
